# Ideological idiosyncrasies and individual-level radicalisation pathways: unpacking the mutating dynamics of contemporary extremism

**DOI:** 10.1080/18335330.2025.2502580

**Published:** 2025-05-08

**Authors:** Jacob Astley

**Affiliations:** University of Liverpool, Liverpool, UK

**Keywords:** Extremism, ideology, pathways, prevent, radicalisation

## Abstract

The rise of ‘mixed’ forms of extremism, particularly esoteric variations that defy conventional ideological boundaries, presents significant conceptual and regulatory challenges. These non-doctrinal forms, characterised by a fluid bricolage of attitudes, beliefs, and identity constructs, transcend existing policy-defined ideological categorisations and pose a complex threat. While extremist phenomena such as inceldom and misogynistic-driven violence have been more recently studied, the broader ecosystem of converging and seemingly disparate threads of extremism remains underexplored. Addressing this gap, this paper illuminates the evolving landscape of radical(ising) pathways and calls for a broadening of radicalisation research to include an exploration of the role and significance of idiosyncratic modes of extremist engagement. It further seeks a deeper understanding of the relational and emotional dimensions of radicalisation, foregrounding the fluid, heterogeneous, and often unpredictable nature of individual-level pathways. By examining these factors, this paper challenges the simplifications embedded in conventional categorisations of ideology and critiques the Prevent Strategy’s capacity to respond to the mutating dynamics of contemporary extremism, arguing for adaptive frameworks that acknowledge and engage with the complex interplay of personalised agency, shifting structural conditions, and the fluid architecture of evolving radical(ising) ecosystems.

## Introduction

Since the creation of the ‘mixed, unstable and unclear’ (MUU) ideology in 2018 for the purposes of categorisation under Prevent – one of the four strands of the UK’s strategic response to terrorism – the way in which it has been constructed, outlined and operationalised has raised a number of acute challenges to scholars. This is unsurprising given that varying modes of extremism have been a prevalent threat in the UK for well over half a century. Although the nature and magnitude of these threats has mutated over time, violent extremism, regardless of the form it takes, remains an omnipresent risk. Whilst the MUU category has been recently re-badged as ‘conflicted’ (Home Office, 2023b), the overarching construct reflects instances where the ideology, as framed by Prevent, is presented as: ‘mixed (involving a combination of elements from multiple ideologies), unstable (shifting between different ideologies), or unclear (where the individual does not present a coherent ideology, yet may still pose a terrorism risk)’ (Home Office, 2018c). Notably, ideologies that have been associated with an MUU identification can include – but are not exclusive to – preoccupations with forms of mass killing, non-ideological hatred of groups and varying degrees of inceldom. One illustrative case is that of Axel Rudakubana, who sadistically murdered three young girls in Southport in 2024. Recently, it has been suggested in hindsight that Rudabukana’s initial Prevent referral in 2019 – when he was just 13 years-old – could have fallen into the MUU category with his ideology categorised as ‘unclear’ (Home Office, 2025). Notwithstanding that Rudakubana was referred to Prevent on three separate occasions – and the concomitant simplistic associations that could be made to the Prevent Strategy’s (in)effectiveness – the case of Rudakubana is emblematic of the growing prominence of three interdependent and currently indeterminable mechanisms: idiosyncratic ideological constructs, esoteric manifestations of (violent) extremism, and the unpredictable nature of individual-level radicalisation.

At the risk of understating some of the historical and contemporary processes that have shaped what is currently understood as ‘extremism’ in the UK, definitions of the key terms in this area are well documented (see HM Government, 2008; 2009; 2011; Home Office, 2011; 2015b; 2018a; 2023a). Critical observations of these developments notwithstanding, what constitutes ‘terrorism’, ‘extremism’ and ‘radicalisation’ are far from universally agreed and remain both contested and contestable concepts. This is particularly significant given that all of these terms have been labile rather than static concepts, leading to the problematic assumption often used to legitimise counter-terrorism measures in British governmental policy rhetoric, that ultimately, extremism acts as a strong feeder pathway into terrorism (Onursal & Kirkpatrick, 2021).

While this discussion uses the UK as a case study to develop a broader conceptual argument about the phenomenon of ‘mixed’ extremism, it is important to recognise that the landscape of contemporary extremist threats in the UK shares many commonalities with other countries across Europe, such as Belgium, Norway, France, and Germany (Europol, 2023). Globally, nations such as New Zealand, Australia, Canada, and the US have similarly experienced acts of terrorism and violent extremism linked to individuals motivated by idiosyncratic ideologies (Meleagrou-Hitchens & Ayad, 2023; Norris, 2020). Concomitantly, the nomenclature of inherently ‘mixed’ forms of extremism has been subject to widespread variation, in part due to the complex nature of enveloping ideologies that blend multiple, diverse and sometimes seemingly contradictory beliefs, rendering them difficult to categorise within traditional frameworks. Nascent terminological conceptualisations include, but are not limited to, salad bar extremism (Wray, 2020), composite extremism (Gartenstein-Ross, Zammit, Chace-Donahue, & Urban, 2023), ideological convergence (Ong, 2020) and idiosyncratic terrorism (Norris, 2020). In terms of governmental strategy, alongside the MUU classification in the UK’s Prevent programme, the US Strategic Framework for Countering Terrorism and Targeted Violence (U.S. Department of Homeland Security, 2019), and the establishment of fixated threat assessment centres in New South Wales, Australia (Farrell, 2017), illustrate some of the broader preventative efforts to tackle the rise of extremist threats that transcend conventional ideological boundaries (Gartenstein-Ross et al., 2023, p. 1). As this discussion later critiques, however, governmental approaches – particularly in the UK – have become compartmentalised, often focusing on various ‘drivers’ leading to the development of extremist worldviews.

This article proceeds by taking a conceptual approach designed to illuminate the changing dynamics of currently manifesting esoteric expressions of contemporary extremism. It extends Norris (2020) concept of idiosyncratic terrorism and Meleagrou-Hitchens and Ayad’s (2023) application of this framework in analysing online mixed extremist communities that converge aspects of Far-Right and Islamist ideologies. Building on this foundation, this contribution aims to break the binary of traditional ambits of extremism, foregrounding the idiosyncratic nature of ideological formations underpinning contemporary forms of extremism which are at once mixed, mutable and inherently esoteric. First, it will probe the contexts that fuel motivations to affiliate with composite extremist beliefs. Second, the opportunities and constraints in which emergent and convergent extremism is developed and operationalised. Third, an assessment will be undertaken of the modes by which socialisation may be transformed into extremist identifications and rationalisations. Overall, it considers how the formation of ideological idiosyncrasies both shapes and is shaped by radical(ising) pathways at the individual-level. Recognising this bidirectional interplay prompts an examination of how ongoing processes, lived experiences and underlying material conditions influence the radical(ising) journeys of individuals. Altogether, this invites a reconsideration of how radical(ising) trajectories can be characterised by the mutation of idiosyncratic ideological constructs and the dynamic evolution of journeys, culminating in esoteric expressions of extremism.

Crucially, although there is a limited comprehensive understanding of the manifold ways in which structural and individual-level processes interact in shaping radicalisation pathways toward extremism (Jensen & Larsen, 2021; Norris, 2020), this contribution seeks to identify several features that both emerge and warrant further exploration. Indeed, whilst significant research has explored the relationship between radicalisation and factors such as, amongst others, group dynamics (Sageman, 2004; 2008) and pathway approaches (Jensen, Atwell Seate, & James, 2020; McCauley & Moskalenko, 2008; 2017), conceptualising and understanding the establishment of mixed and mutable extreme worldviews requires acknowledging wider complex and context-dependent factors beyond pure ideological attraction (Adams, Stahl, & Oberg, 2023). Whilst this contribution does not seek to explicitly critique traditional models of radicalisation *per se*, it does call for the expansion of radicalisation research to include those influenced by idiosyncratic ideological constructs. Specifically, excavating the interplay between complex personal subjectivities, social and/or collective identities and ontological (in)security, which underpin the potential influence of radical(ising) spheres that may facilitate the formation of contemporary extremist views (see Ferguson & McAuley, 2021; Pilkington, 2023). Running parallel, there is limited appreciation of the mechanisms and processes through which psychosocial traits may alter the worldview of individuals who may already be socially vulnerable in diverse ways and how this connects with expressions of identity (see Whiting, Spiller, & Awan, 2024). Ultimately, unravelling the complex and mutating ways in which these factors affect individual agency and shape worldviews, as well as recognising the varied properties of contemporary threats which elude traditional categorisations, is crucial in understanding the heterogeneous radical(ising) pathways into extremism that individuals may take.

## Contested terms and processes: extremism and radicalisation

Prior to outlining the challenges that the UK has faced both in defining and conceptualising extremism and radicalisation, it is important to first clarify the key terms. Despite the original policy document for the UK’s counter-terrorism strategy – known as CONTEST – being first released to the public in 2006 (HM Government, 2006), spawning a series of later iterations (HM Government, 2011; Home Office, 2018a; 2023a), it was not until 2011 that a formal definition for extremism was advanced which defined it as ‘vocal or active opposition to fundamental British values, including democracy, the rule of law, individual liberty and mutual respect and tolerance of different faiths and beliefs’ (Home Office, 2011). Although not designated as statutory until the passing of the Counter-Terrorism and Security Act (2015), various viewpoints have been expressed about its usefulness. Notably, that by being defined this way it has generated tangible interpretational challenges and highlighted latent issues that the underlying processes are vague, over expansive, gender-blind and difficult to apply in practice across institutional settings (Aked, 2020; Kundnani, 2014; Lowe, 2017; McCulloch, Walklate, Maher, Fitz-Gibbon, & McGowan, 2019). While the above attests to a landscape of conceptual and operational confusion, this onerous task has been recently reignited by a fresh attempt to define ‘extremism’ by the UK Government. Aside from the many underlying reasons that necessitated an update to the definition since it was first set out in 2011 – allied in part to the recognition of an ‘ever-evolving threat’ – the new definition portrays extremism as:

‘The promotion or advancement of an ideology based on violence, hatred or intolerance, that aims to:

1. Negate or destroy the fundamental rights and freedoms of others; or
2. Undermine, overturn or replace the UK’s system of liberal parliamentary democracy and democratic rights; or
3. Intentionally create a permissive environment for others to achieve the results in (1) or (2).’

(Department for Levelling Up, Housing & Communities, 2024).

Although perhaps impetuous to assess the impacts and implications of the updated extremism definition given its newness, there are some elements that require unpacking. In particular, the new definition brings to the fore a range of salient issues around the deployment of technologies of ‘risk’ in identifying potential extremist threats. Concomitantly, suggestions also point to a failure to allay long-standing fears associated with the old definition of extremism – namely, the disproportionate targeting of Muslim communities (Badshah, 2024). This can be exemplified by the treatment of CAGE and MEND, both of which serve as advocacy groups who aim to raise awareness and empower British Muslim communities. In recent years, these organisations have come under the spotlight for allegedly fostering grievances related to societal (in)security and isolation, partnering with actors perceived to pose ‘extremism’ concerns, and advancing so-called extremist ‘anti-Prevent’ narratives thought to contribute to the spread of mis- and disinformation (see Shawcross, 2023). Particular reference has been made to a number of publications by both groups (see CAGE, 2016; 2021; MEND, 2017; 2023). Nevertheless, comments made by the then Secretary of State for Levelling Up, Housing and Communities, Michael Gove, asserting that organisations such as CAGE and MEND ‘give rise to concern because of their Islamist orientation and views’ (Hansard HC Deb., 14 March 2024) raise significant considerations. Such statements risk perpetuating erroneous assumptions that Islamic symbolism inherently signifies affiliation with extremist ideals and/or groups, or indicates an increased risk of radicalisation – the deleterious impacts of which are well documented (see Abbas, 2019; Kundnani, 2014; Mythen, Walklate, & Khan, 2009). The designation of these organisations on a ‘watchlist’ further signals a broader concern, that those who may choose to criticise government and State institutions run the risk of being labelled ‘extremists’, thereby criminalising legitimate forms of ideological opposition and political dissent.

Here, it is worth noting that the UK government has a history of misinterpreting who and what meets the threshold to be considered extremist. A pertinent example is the misclassification of Extinction Rebellion (XR), an environmental movement advocating for systemic change through non-violent civil disobedience, which was controversially listed alongside proscribed extremist groups such as National Action and Al-Muhajiroun by Counter Terrorism Policing South East (Dodd & Grierson, 2020). Crucially, XR’s motivations centre on challenging the fundamentally destructive relations of modern Western society through an evolving concept termed ‘regenerative culture’ (Extinction Rebellion, 2023; Westwell & Bunting, 2020). Perhaps unsurprisingly, the inclusion of XR was dismissed and clarified as a mistake. This, however, only augments the deleterious impacts and implications problematising precisely what denotes ‘extremism’ and an ‘extremist’ organisation. While such critiques of macro-level attempts to problematise, even criminalise, legitimate oppositional discourses and the silencing of political dissent have been well noted (see Mythen, Walklate, & Khan, 2013; Skoczylis & Andrews, 2021), it is clear that there are lessons yet to be learned. Not least since these manifold issues arguably represent only the thin end of the wedge. Amidst a landscape of ideological idiosyncrasy, it is critical to recognise that extremism does not exist in a vacuum. Failure to acknowledge this risks not only normalising the conflation of lawful dissent with extremism and entrenching public mistrust, but also risks diverting attention from emergent and evolving threats, consequently reinforcing perceptions that Prevent’s priorities remain misaligned and disproportionately focused.

Having probed ongoing debates around the nature and meaning of extremism, radicalisation – both as a concept and as a set of processes – has also proven to be the subject of widespread debate in media, academic and political circles (see Cottee, 2023; Kundnani, 2012; Mythen, Walklate, & Peatfield, 2017). Although it must be borne in mind that any policy that attempts to *prevent* radicalisation will inevitably be subjected to scrutiny and potential criticism, early UK governmental policy adopted the discourse of a ‘radicalisation process’ (HM Government, 2006; 2009). The implications of framing radicalisation as a ‘process’ – often associated with the widely criticised ‘conveyor belt’ model – have been critically highlighted for relying on nebulous definitions and classifications that create dangerous scope for misinterpretation (Coolsaet, 2023; Mythen et al., 2017; Walklate & Mythen, 2015). Although UK radicalisation prevention strategies have the capacity to produce risk mitigation practices – as evidenced in the Channel process – the broader framing of radicalisation as the sum of pre-established, determinable factors, wherein key moments leading to the adoption of extremist values are compartmentalised, has long been considered an albatross around the Prevent Strategy (Heath-Kelly, 2013; Mythen et al., 2017). Indeed, it runs counter to the plethora of work by scholars – of which this contribution highlights just two prominent frameworks – that have reinforced the need for better conceptualisations of radicalisation and stress its inherently non-linear nature (see Khalil, Horgan, & Zeuthen, 2019; McCauley & Moskalenko, 2017).

Moreover, the importance of non-linear pathways is not a novel one in radicalisation literature. For instance, the work of Borum (2011a; 2011b), who in reviewing a number of different frameworks and models – from social movement theory (see Wiktorowicz, 2005), social psychology (see McCauley & Segal, 1987) and conversion theory (see Lofland & Stark, 1965), emphasises that radicalisation should be viewed as a set of dynamic processes that happen in the real world. In doing so, he argues that ‘different pathways and mechanisms of terrorism involvement operate in different ways for different people at different points in time and perhaps in different contexts’ (Borum, 2011a, p. 7). Notably, this principle of multifinality – just one of the insights on which there is growing consensus in radicalisation research (see Dawson, 2019) – has recently been applied in the context of vulnerability indicators of lone-actor terrorism. For Corner, Bouhana, and Gill (2019), it is essential to disaggregate the concept and construct of ‘vulnerability to radicalisation’ to recognise the distinct dimensions of both ‘vulnerability’ and ‘vulnerability to radicalisation’. This distinction becomes particularly pertinent when recognising that lone-actor radicalisation pathways have been considered individualised, relational processes (Lindekilde, Malthaner, & O’Connor, 2018; Malthaner & Lindekilde, 2017). Viewed in this light, lone-actor radicalisation transcends the conceptual simplicity of social isolation emphasised in early work on the phenomenon (see McCauley & Moskalenko, 2014), instead hinging on socialisation processes and patterns shaped by individual subjectivities and consolidated by situational and affective factors (O’Connor, Lindekilde, & Malthaner, 2023).

Whilst now is not the time to become ensconced in a discussion of lone-actor radicalisation, its brief inclusion underscores the idiosyncratic ways in which lone-actors espouse extremist attitudes and beliefs (see Hamm & Spaaij, 2017), a key element in understanding the mutating dynamics of radicalisation pathways. Returning to the quandaries surrounding the challenges faced by de-radicalisation strategies, the discussion turns to a number of prescient concerns associated with the Channel programme – the UK’s approach to radicalisation risk mitigation. Promoted as a multi-agency initiative, Channel panels are led by the police and chaired by a local authority representative, with participation from professionals such as youth workers, social workers, doctors, and religious leaders. The programme's stated objective is to protect vulnerable people by identifying potential harm, assessing the nature and extent of risk, and developing appropriate mentorship and support plans for individuals at risk (Home Office, 2015a). Despite generating comparisons with risk mitigation measures from counter-terrorism agencies within Europe, such as Denmark and its promotion of a mentoring approach known as the ‘Aarhus model’ (see Bertelsen, 2015), the validity and reliability of the Channel programme have been called into question (see Mythen & Baillergeau, 2021). Particularly as a result of numerous dubious cases including a University of Staffordshire student, Mohammed Umar Farooq, who was flagged for reading textbooks on terrorism; a sixteen year-old for taking out a library book on terrorism; and a four year-old who mispronounced cucumber as ‘cooker-bomb’ (Lowe, 2018; Mythen et al., 2017). In such instances, what may ostensibly appear to be common elements of adolescent journeys have the capacity to be institutionally recast as ominous risk factors, potentially problematising and criminalising processes associated with adolescent socialisation (Mythen, 2020; Mythen & Baillergeau, 2021). While sensationalised media stories of questionable referrals have been noted to heighten public attention to cracks within the Prevent system (Thomas, 2020), equally critical – and arguably more disquieting – are the immediate and longer-term iatrogenic effects of ‘false positives’ on individuals, their families, and the wider community (Aked, 2020).

Suffice it to say at this juncture, the new definition of extremism is far from constituting a panacea. Indeed, although it seems axiomatic that policies designed to *prevent* the processes of radicalisation should be evidence-based, strategies such as Prevent and Channel have become synonymous with erroneous assumptions. Moreover, recent efforts to bolster the definition of extremism and avoid reverting to the ‘conveyor belt’ model of radicalisation perennially associated with the Prevent Strategy have only exacerbated extant anxieties and reignited questions of whether risk-based decision-making can inform appropriate interventions and assuage the (re)production of iatrogenic effects (Mythen, 2020; Mythen & Baillergeau, 2021; Pilkington & Acik, 2020). Alongside such definitional efforts, a further salient consideration comes to the fore. Conceptually, such efforts to re-evaluate extremist threats have failed to dynamically respond to the increasing fluidity of ideological idiosyncrasies. Ostensibly, this critical misalignment between the Prevent Strategy’s structural rigidity and the adaptive, often transient affiliations of modern-day actors can be exemplified by the strategy’s inadequate response to the unique challenges posed by individuals whose radical(ising) influences are highly personalised and mutable – an issue to which this discussion now turns its attention toward.

## Conceptualising and understanding ‘mixed’ ideological currents

As the global context of extremism rapidly evolves, so too does our understanding of its nature. Against this backdrop, are the relational dimensions of newly emergent extremism. This lens emphasises how such dynamics contribute to the mutating radicalisation pathways central to this contribution’s argument. Specifically, it considers the creation and operationalisation of the MUU category, which encompasses individuals who amalgamate aspects of multiple ideologies and/or alternate between them without fixed alignment. According to the Home Office (2018c, p. 8), the impetus for introducing the category stemmed from ‘exploration and development of the statistics, and a genuine increase in the number of cases presenting with these kinds of ideology’. It replaced the former ‘unspecified’ category in previous iterations of the annual Prevent statistics, under which only 11% (696 out of 6,093) of individuals received an initial referral, and none were discussed at a Channel panel or adopted as official Channel cases (Home Office, 2018b). Although it is important to avoid casual associations between the ambiguously defined ‘unspecified’ label and its (potential) facilitation in enabling individuals to slip the net, the recorded proliferation of cases falling under MUU accentuates the need to unpack the intersecting and converging characteristics of contemporary extremist threats.

That individuals who fall under the MUU category both elude traditional categorisations of extremism due to the fractionalised nature of beliefs and represent the largest and fastest growing group referred to Prevent is axiomatic. The decision then announced by the Home Office (2023b) to replace the MUU category with a new catch-all category dubbed ‘conflicted’ appears arcane. Aside the extent to which this sudden change adequately captures the diverse manifestations of extremism embedded within MUU – including those who are, amongst others, militant, misogynistic, anti-government or support conspiracy theories – the pattern remains constant. The majority of recent Prevent referrals are not classed as either Islamist or Far-Right (see Home Office, 2024). This observation arises at a critical juncture, amid assertions that Prevent has suffered a ‘loss of focus’ on Islamist extremism have been erroneously attributed to the proliferation of MUU referrals, which are believed to have caused a misalignment of the strategy’s priorities (see Shawcross, 2023). The proposed rectification – the wholesale removal of the MUU category – is framed as a means to cure Prevent, which has supposedly been rendered ‘out of kilter’. Whilst a deeper critique of the ‘independent’ review of Prevent lies beyond the scope of this discussion, it is clear that the review reasserts Islamist extremism as the perceived ‘real risk’ in the UK. More broadly, it reflects a reluctance to conceptualise and confront the growing trend of individuals espousing dangerous ideas rooted in disparate values and ambivalent ideologies, often marked by internal incoherence or uncertainty. It is also worth contemplating whether – and to what extent – the structures and processes underpinning Prevent are capable of addressing contemporary engagement with more diffuse forms of extremism that may lead to indiscriminate violence across a range of public and private contexts. To this end, it is imperative that the Prevent Strategy evolves to address emergent and convergent potential threats that manifest as mixed and mutable typologies of extremism. To supplement this, the strategy must also seek to elucidate a more granular understanding of individual radicalisation processes, fostering an approach that is as mutable as the worldviews it seeks to combat.

Having drawn attention to recent shortcomings of policy-making, the critical importance of advancing a more robust conceptualisation of fluid, fragmented and idiosyncratic ideological currents should not be understated. Whilst the current body of research on MUU extremism – and, by extension, ideological currents that are inherently ‘mixed’ – remains sparse, emerging work indicates that the incitement of hatred and violence along socio-political and ideational lines are salient considerations (Brace, Baele, & Ging, 2024; Gartenstein-Ross et al., 2023; Meleagrou-Hitchens & Ayad, 2023). For instance, commonalities have been noted between anti-immigrant, incel and radical masculinist groups who draw from different ideological repertoires, but who rationalise violence as a means of provoking socio-political change and restoring a perceived threatened social order (Rousseau, Aggarwal, & Kirmayer, 2021). While a number of proximate incidents and conflicts have informed the expansion of risk prevention and mitigation strategies in the UK, recognising the growing diversification of belief systems, identity constructs and associated grievances – beyond the traditional binary – is essential to grasping the interconnected dynamics of emergent extremism. Not least the MUU category, which demands a more nuanced understanding capturing both ideological fluidity and the specific radical(ising) processes through which individuals engage. Ultimately, these ideological fragments do not exist in isolation, but are moulded within broader ecosystems of grievance and meaning, shaping unpredictable individual pathways into extremism.

This shift accentuates the need to reconsider conventional frameworks and aligns with this contribution’s focus on conceptualising the bidirectional interplay between ideological idiosyncrasies and individual-level radicalisation pathways. This imperative emerges amid the growing number of individuals engaging with material drawn from disparate threads of extremism that extend beyond the traditional ambits of Far-Right and Islamist ideologies. Extensive research examining incels and the broader, interconnected online subcultures of men’s marginalisation, aggrievement and vitriolic misogyny testifies to this trend (see Baele, Brace, & Coan, 2021; Ging, 2019; Hoffman, Ware, & Shapiro, 2020; Sugiura, 2021; Tranchese & Sugiura, 2021). Previous work by Agius et al. (2020) has particularly shown that online discourses emerging from within the manosphere and adjacent Far-Right ecosystems reinforce emotional aggrievement, provide justification for violence-supportive attitudes, and offer a sense of belonging and ideological coherence not typically found in other spaces. Yet, there remains limited research on the radicalisation processes within the manospshere (see Botto & Gottzén, 2024; Regehr, 2022). For Botto and Gottzén (2024) in particular, their focus on radicalisation pathways in the manosphere has found ideological and psychosocial dynamics to be salient considerations, especially the ways these intersect with young men’s self-identified sense of vulnerability and aggrievement as perceived victims. This is further underscored by Baele, Brace, and Ging (2023), whose diachronic cross-platforms analysis of the ‘incelosphere’ – identified as the most extreme manosphere subculture – emphasises that the formation of extreme worldviews, marked by dynamism and heterogeneity, unfolds within an ideational landscape that shapes itself as a continually evolving ecosystem.

What materialises, then, is the notion that individuals are not necessarily attracted to extremism solely for ideological reasons. Rather, selective ideological references often serve as entry points into online echo chambers, which intersect with wider experiences of ontological insecurity. At a broader level, this can be understood by eliciting the routes and vehicles through which problematic values and beliefs emerge and evolve, and by unravelling the extent to which engaging with and espousing mixed and mutable extremist viewpoints can be indexed to certain material conditions and lived experiences. While previously understood that top-down structures permitted extremist formations to develop and become attractive to socially marginalised individuals, increasing recognition is being given to the role of organisational decentralisation (see Europol, 2023; Gartenstein-Ross et al., 2023). Keying into this is the concept of ‘ideological convergence’ (Ong, 2020) – one of the nascent terms variously used to describe inherently ‘mixed’ ideological formations. Observations of its usefulness and utility notwithstanding, two interconnected insights stem from this term. First, that social polarisation – which results, at least in part, from violence between in- and out-groups – serves as a catalyst for convergence; and second, that the absence of a guiding hierarchical organisational structure engenders a culture of ‘cherry-picking’ to suit an individual’s subjective worldview (Ong, 2020). In some regards, this may well be a conflation of existing ideological currents, yet it may also constitute an expressive bricolage of loosely aggregated ideological motifs rather than a coherent hybrid. For instance, particular proclivities such as antisemitism that undergird a myriad of ideologies have been suggested to facilitate the adoption of and migration between multiple – and not always intersecting – ideological positions (Gartenstein-Ross et al., 2023).

Accordingly, ideological fragmentation appears to be a natural extension of organisational decentralisation. This dynamic has the potential to give rise to esoteric permutations of extremism that defy simple categorisation due to their emergence within decentralised, horizontally networked spaces of communication. This notion assumes greater significance when considering that the definitive objectives of well-established extremist groups have been increasingly supplanted by individuals whose engagement is characterised by labile affiliations and amorphous aspirations. Such individuals are often influenced by loosely associated ideological motifs, facilitating narrative personalisation and the construction of bespoke forms of extremism. Ultimately, it prompts this discussion to consider the etiology of radical(ising) journeys and trajectories therein, and the ways in which individuals may essentialise fragments of meaning to rationalise their radicalisation in response to their lived experiences and situational worldviews.

## Reimagining radicalisation journeys: toward individual-level pathways?

Reimagining radicalisation journeys to reflect the esoteric nature of contemporary extremist engagement requires acknowledging that a number of seemingly disparate actors now populate increasingly convergent spaces. Notwithstanding significant empirical and theoretical contributions demonstrating that collective radicalisation is inherently relational – shaped by interactions between individuals, within movements or groups, and consolidated through emotional bonds (see Bosi, Demetriou, & Malthaner, 2014) – it is the evolving nature of these relational processes that warrants further examination. Central to this evolution is the imperative to bridge the micro–macro divide. Foregrounding the relational interplay between micro-level mechanisms and macro-level conditions offers a productive way to refine theoretical approaches to understanding radicalisation (see Jensen & Larsen, 2021). For these scholars, this perspective is bolstered by an appreciation of the role of religious emotions in Islamist radicalisation, which constitute a fundamental component of relational radicalisation processes. Crucially, recognising that emotions are not static but inherently dynamic allows for a deeper exploration of the extent to which they may be (trans)formed – for instance, in response to the pursuit of ontological security, experiences of emotional reciprocity (see Pilkington, 2023) and/or alignment with identity (trans)formation (see Munden, 2025).

While research has primarily focused on emotional dimensions within Islamist and Far-Right contexts, a critical lacuna remains – one in which studies could apply similar approaches when considering the idiosyncratic ideological constructs emerging within contemporary radical(ising) milieus. Recent work by Gartenstein-Ross and Blackman (2022, p. 555), for instance, has conceptualised ‘fringe fluidity’ as a radicalisation pathway where individuals ‘transition from the embrace of one form of violent extremism to another’. Central to this is the idea that ‘fringe fluidity’ is not simply a process of inter-ideological borrowing and sharing that facilitates a congruence of extremist perspectives, but is, in fact, its own distinct individual-level radicalisation pathway (Gartenstein-Ross & Blackman, 2022). This stands in contrast to earlier conceptualisations of individual-level radicalisation that have, to some extent, become compartmentalised, often focusing on how relatively normal people come to accept and act upon extremist beliefs that advocate violence (see Malthaner, 2017). While ideological beliefs are not causative drivers of behaviour, they nonetheless remain significant insofar as they provide individuals with a framework through which to make sense of – and communicate – their internal narrative logic (Adams et al., 2023). Ostensibly, complex and context-specific engagement both with and within radical(ising) milieus may enable individuals to transcend a deeply troubled and damaged sense of self – one that exists outside the confines of dominant binary conceptions of extremism.

What can be more conclusively suggested, then, is the need to consolidate efforts to excavate the latent factors that foster ideational intrigue and interaction. Whilst far from concretised at present, there is a risk in attempting to objectively disaggregate such ‘drivers’ given the inherently fluid and subjectively curated nature of personalised belief systems and identity constructs. This approach, however, only becomes reductionist if ideology continues to be framed as a necessary endpoint. The critical problems and challenges which undergird this onerous task, require us to consider that such explanatory factors, which may serve as entry points and accelerators within radical(ising) spheres, derive significance not from their directional push or pull toward an ideological framework, but from the ways they shape subjective experiences of marginalisation, injustice and/or alienation (Adams et al., 2023). In overlooking the emergent dynamics that challenge conventional understandings of radicalisation, and the deeper shifts in how contemporary extremist expression transcends ideological alignment, we risk misidentifying and failing to address idiosyncratic and esoteric manifestations that elude our conceptual frameworks – pushing us toward an epistemological crisis.

This is particularly relevant to the mutating dynamics of contemporary extremism posited in this contribution, where radicalisation journeys defy fixed ideological affiliations and instead reflect individualised processes and idiosyncratic forms of interaction. Such observations necessitate a reconsideration of how the micro–macro dialectical relationship functions in fostering esoteric manifestations of extremism. It further elucidates that there is an intricate interplay between ideational and ideological radicalisation and the significant roles of the relational and emotional dimensions. Specifically, the crucial role that radical(ising) spheres play in shaping the processes and patterns of socialisation – both of and between individuals – and how they contribute to satisfying an individual’s purpose, expression and action (Malthaner & Waldmann, 2014; Pilkington, 2023). This becomes especially pertinent when we consider that evolving online ecosystems further intensify these dynamics by engendering new modes of socialisation, exposing individuals to extreme narratives and facilitating anomalous interactions and patterns of engagement (see Williams & Tzani, 2024). Here, it is equally critical to acknowledge – and continue to challenge – the false dichotomy between offline and online radicalisation, recognising that these realms are deeply intertwined and mutually reinforcing (Valentini, Lorusso, & Stephan, 2020). Altogether these insights underscore the need to revisit arguments that foreground an individualised process, wherein specific moments introducing radical ideals can potentially trigger ‘cognitive openings’ (see Wiktorowicz, 2005), thus transitioning individuals from ‘opinion radicalisation’ to ‘action radicalisation’ (see McCauley & Moskalenko, 2017).

In advancing our understanding of cognitive openings, recent work has stressed the need to better conceptualise what may act as ‘triggers’ (Munden, 2025). This is particularly relevant, for example, in the context of young men and boys who have been observed to seek out alternative narratives, fragmented ideological currents, and social possibilities in an attempt to make sense of their identity, beliefs, behaviour and, ultimately, where they belong in the world (Nicholas, 2024). That we know these factors underpin the mechanisms of rationalisation potentially leading individuals toward adopting extremist identities only reinforces the need for a more nuanced understanding of how psychosocial and fragmented ideological motifs converge to influence radicalisation journeys. Despite these forward steps, however, translating these critical arguments to the journeys of individuals espousing inherently ‘mixed’ ideological fragments – which manifest as esoteric expressions of extremism – remains underutilised.

One way this might be achieved is through the recently conceptualised theoretical Cognitive-Emotive Model of Radicalisation. Advanced by Howard, Poston, and Lopez (2024), this model is utilised to explore how neurocognitive mechanisms underpinning radicalisation – particularly in online contexts – can arouse empathy and permit individuals to transform from a passive consumer of extremist narratives into an active participant with a radicalised mindset. It further foregrounds an individualised process and the saliency of their curated identity construct and belief system, thereby reinforcing the integral roles of the relational and emotional dimensions of radicalisation. Crucially, not only can emotional engagement be stimulated to shape an individual’s attitudes and beliefs, but exposure to radical(ising) content across the wider radical(ising) ecosystem can also trigger cognitive and affective responses that facilitate existential worldview shifts. This notion of ‘turning points’ or trajectories – as first exemplified by Horgan’s (2008) argument to replace searching for ‘roots’ of violent extremism in favour of understanding ‘routes’ – presents a favourable avenue. For Pilkington (2023), it provides a conceptual tool to capture the complex and changing engagement of individuals both with and within radical(ising) milieus, allowing for the identification of important transitions and the underlying lability of radicalisation journeys. Intrinsically, this approach recognises the heterogeneous nature of potential pathways into and within extremism, and acknowledges that the complex interplay between individual subjectivities and shifting ideological currents gives individuals latent capacity and agency.

All of this appears to support the view that the role and significance of ideology in radicalisation journeys into extremism is variable. It also warns against gravitation toward ‘one size fits all’ answers. This, at a time when recent attempts have been made to reinforce this notion within the UK’s ‘independent’ review of Prevent: ‘I [Shawcross] am forced to raise the question of whether it makes sense to refer to Prevent individuals who have no clear ideology’ (Shawcross, 2023, p. 53). Whilst questions about the relative significance of ideology are likely to rumble on, what materialises is a lack of understanding of ideological fragmentations and the migration towards post-organisational trends. Indeed, although almost all extremist ideology is regarded as extreme relative to the government’s position, the esoteric nature of ideological idiosyncrasies raises acute challenges as to what counts as a single discernible ideology and whether it should be attributable to an extremist threat. As Meleagrou-Hitchens and Ayad (2023) argue, while many online, idiosyncratic communities may appear ‘mixed, unstable, or unclear’, they are deeply rooted in ideological principles that predate their presence online. Thus, recognising and addressing newly emerging and converging ideological currents, which often begin circulating in wider online ecosystems long before they are attributed as potentially causative to an act of extremist violence, presents a logical way forward. Ultimately, this offers one potential avenue to ameliorate the shortcomings of ‘policy-based evidence-making’ (see Mythen et al., 2017, p. 196), by advancing a conceptual lens attuned to ideological idiosyncrasies and the intricacies of narrative personalisation – one that engages with the complex and mutating nature of individual agency that gives rise to distinct individual-level radicalisation pathways.

## Conclusion

Against a backdrop of increasing social polarisation, what emerges is not a predictable progression from belief to action, but a fluid bricolage of curated fragments, shifting emotional alignments, and mutating personal narratives – each individual a distinct expression of radical(ising) agency shaped within, but not determined by, the wider radical(ising) ecosystem. That extremist views are not uniformly held even among like-minded individuals, across adjacent spaces, or at any given time, further reinforces the need to conceptualise contemporary extremism as esoteric. In turn, radicalisation processes are evolving, reflecting a move away from the top-down structures of established groups toward horizontal, individualised networks. Taken together, these observations underscore the critical interdependence between mutating worldviews and radical(ising) processes, wherein shifts in one can precipitate changes in the other. This dynamic interplay necessitates a comprehensive re-evaluation of the complexities of contemporary mechanisms and underpinnings of radicalisation, to avoid falling into an epistemological crisis that may further obfuscate our understanding. Thus, what must now be recognised is the symbiotic relationship between ideological idiosyncrasies and the radicalisation processes they engender, which has facilitated the emergence of multifarious and intersecting radical(ising) avenues through which inherently esoteric expressions of extremism can flourish.

The recent trend of subsuming counter-extremism policies within the broad-brush framework of the UK’s counter-terrorism strategy comes at a time when prevailing anxieties about the principal threat of extremism appear misguided and the mutating radical(ising) landscape is overlooked. Although it has been suggested that the adoption of an inherently ‘mixed’ ideology may function as a rationalisation for violence rather than a ‘driver’ (see Comerford & Havlicek, 2021), this would require moving beyond reliance on broad conventional ideological categories. Accordingly, this necessitates revisiting models that attend not only to the ideological influences on the radicalisation of opinion, but to the relational, affective and contextual mechanisms that may incline individuals toward radicalisation of action (see Jensen & Larsen, 2021; McCauley & Moskalenko, 2017). At the same time, there is a risk in essentialising the ‘drivers’ of extremism as rooted solely in specific ideologies, which in turn risks obscuring the wider constellation of psychosocial and context-dependent factors that shape radical(ising) pathways (Adams et al., 2023; Gill, Horgan, & Deckert, 2014). Yet, there is an equal risk that entirely dismissing the role of ideology becomes reductive. This is particularly relevant when considering the increasing lability of individuals within movements and subcultures who extract fragments from multiple, disparate ideological repertoires that are neither necessarily mutually consistent, nor indicative of firm adherence. Such fragments scaffold the construction of idiosyncratic extremist rhetorics – ones that seek to justify, symbolise and/or express deeply personalised worldviews. Altogether, this reflects how the processes of radicalisation are influenced by the unpredictable confluence of a number of intersecting, granular factors that underpin both the formation of individual-level pathways and the subsequent evolution of journeys.

Aside from the paucity of knowledge presented by the Prevent Strategy regarding processes of radicalisation, its continued framing as a tangible, deterministic process not only runs counter to contemporary trends but reflects a myopic approach to tackling the evolving spectrum of threats in the UK and beyond. At a time of ongoing definitional and conceptual confusion about how to assess and respond to the broadening gamut of extremism-related challenges, this exemplifies the need to reimagine radicalisation journeys and consider the ideational, affective and situational factors that influence personalised proclivities to engage with extremism. Acknowledging the interrelation between individual subjectivities and identities; how individuals interact with and experience the wider radical(ising) ecosystem; the significance of agency – including whether individuals are passive or active agents – are all of paramount importance in advancing future research in this nascent and rapidly evolving field.

## Data Availability

Data sharing is not applicable to this article as no new data were created or analysed in this study.
